# Neurite orientation dispersion and density imaging identifies microstructural alterations in patients with cerebral microbleeds and correlations with cognitive impairment

**DOI:** 10.1186/s12883-026-04994-3

**Published:** 2026-05-23

**Authors:** Fang Li, Qili Hu

**Affiliations:** Department of Imaging, The Fifth People’s Hospital of Shanghai, Fudan University, No.128 Ruili Road, Minhang District, Shanghai, 201100 China

**Keywords:** Cerebral Microbleeds, Neurite Orientation Dispersion and Density Imaging, Caudate, Cognitive impairment

## Abstract

**Background:**

Neurite Orientation Dispersion and Density Imaging (NODDI) has advanced the study of cortical microstructure. However, its application to participants with cerebral microbleeds (CMBs) to explore cognitive impairment mechanisms and potential imaging biomarkers remains underexplored.

**Methods:**

This observational study used NODDI to assess cortical microstructural changes in three groups: 52 participants with cerebral small vessel disease and CMBs (CSVD-c), 78 CSVD participants without CMBs (CSVD-n), and 37 healthy controls. Multivariable regression analysis examined the correlation between altered NODDI metrics, CMB severity, and cognitive function. Cox regression analysis evaluated the association between altered NODDI metrics and dementia risk.

**Results:**

The CSVD-c group showed a significant decrease in Intracellular Volume Fraction (ICVF) in the bilateral caudate nucleus and an increase in Isotropic volume fraction (ISOVF) in the right precuneus compared to both the controls and CSVD-n groups. Decreased ICVF in the bilateral caudate nucleus correlated with the number of CMBs (*P* = 0.023), lower logical memory scores (*P* = 0.003), and higher Rey Auditory Verbal Learning Test and Clinical Dementia Rating-Sum of Boxes scores (*P* = 0.011; *P* < 0.001). Increased ISOVF in the right precuneus was significantly associated with a higher risk of dementia (*P* = 0.039), but this significance was lost after adjusting for age, sex and years of education.

**Conclusion:**

Participants with CMBs exhibit decreased ICVF in the bilateral caudate nucleus and increased ISOVF in the right precuneus, associated with cognitive impairment and increased dementia risk. NODDI metrics may serve as valuable markers for investigating CMB-related cognitive impairment.

**Supplementary Information:**

The online version contains supplementary material available at 10.1186/s12883-026-04994-3.

## Introduction

Cerebral microbleeds (CMBs) are small, rounded, hypointense foci < 5 mm, occasionally ranging between 5 and 10 mm, detectable using T2* sequences or susceptibility-weighted imaging [1, 2]. Histopathologically, CMBs are focal deposits of hemosiderin-laden macrophages [3]. CMBs have been considered as an important neuroimaging feature of cerebral small vessel disease (CSVD), frequently co-occurring with subcortical infarctions, lacunes, white matter hyperintensities (WMHs), and enlarged perivascular spaces (EPVSs) [4]. The occurrence of CMBs serves as an independent risk factor for intracranial hemorrhage [5, 6].

Recent studies have highlighted the association between CMBs and decreased cognitive performance in CSVD patients. CMBs are reported in approximately 25% to 33% of patients seen in memory clinics [7, 8]. Comparative studies of various cognitive disorders reveal the high prevalence of CMBs in vascular dementia (59%), alcohol-related dementia (40%), and unspecified dementia (33%) [8]. In both general and high-risk populations, CMBs are linked to an increased risk of future dementia [9]. This is consistent with neuropathological studies in elderly individuals, indicating that CMBs are associated with increased odds of Alzheimer’s disease dementia and a more rapid decline in cognitive functions, even after adjusting for other common age-related neuropathologies [10].

However, studies on the neural mechanisms of CMB-related cognitive impairment and its diagnostic and therapeutic markers are limited. A systematic review failed to find a correlation between CMB number or location and neuropsychological profiles in Alzheimer’s disease [11]. Beaman et al. discovered that superficial CMBs are associated with larger morphometric brain measures, particularly white matter volume [12], suggesting that CMBs’ contribution to neurodegeneration may not involve tissue loss. Diffusion-weighted imaging, which measures water molecule diffusion within brain tissues, offers greater sensitivity than traditional T1-weighted imaging for detecting subtle cortical changes. Sui et al. further utilized DWI-based diffusion tensor imaging (DTI) and found extensive white matter microstructural deterioration in CSVD patients with CMBs. Combining DTI-derived diffusivity and anisotropy metrics provides complementary information for assessing white matter alterations linked to cognitive dysfunction, serving as a potential discriminative pattern to detect CSVD at the individual level [13].

Compared to traditional well-established DTI models, which measures variations in the magnitude or directionality of diffusivity but tends to oversimplify diffusion processes and cannot distinguish underlying biological processes. Multi-shell DWI acquisitions and sophisticated multi-compartment modeling techniques, such as Neurite Orientation Dispersion and Density Imaging (NODDI), have markedly improved our capacity to study and characterize cortical microstructure in detail [14, 15]. NODDI, grounded in a biophysical framework, analyzes brain changes in specific microstructural environments. It differentiates the DWI signal from three distinct compartments: intraneuronal (within neurites), extraneuronal (outside neurites), and cerebrospinal fluid (free water). The primary NODDI metrics include the neurite density index (ICVF), quantifying the signal fraction within neurites; the orientation dispersion index (ODI), reflecting angular variation among neurites; and the isotropic volume fraction (ISOVF), indicating the signal fraction from the free water compartment. NODDI has been applied to various central nervous system diseases, including multiple sclerosis [16], Parkinson’s disease [17], and Alzheimer’s disease [18], and is considered a valuable imaging method. However, its application to CMBs remains unexplored.

This study aims to explore the mechanisms underlying cognitive impairment related to CMBs and to identify potential neuroimaging markers using advanced NODDI technology. In addition, we will compare traditional imaging markers with NODDI metrics to evaluate their relative effectiveness. We hypothesize that diffusion MRI technology utilizing the NODDI model will reveal distinct cerebral microstructural damage locations in individuals with CMBs compared to those with CSVD without CMBs and healthy controls. These NODDI metrics are expected to be more correlated with CMB severity and cognitive impairments in participants with CMBs than traditional imaging metrics.

## Results

### Participant characteristics

The study included a total of 167 participants, categorized into three groups: 37 healthy controls (HC), 78 participants in the CSVD-n group, and 52 participants in the CSVD-c group. There were no significant differences in age, sex, or years of education among the HC, CSVD-n, and CSVD-c groups (Age: *P* = 0.144; Sex: *P* = 0.269; Years of education: *P* = 0.743). Regarding CSVD imaging markers, except for the presence rate of CMBs, there were no significant differences in other CSVD characteristics including the number of lacunes, the presence rate of moderate-to-severe EPVS, and the presence rate of moderate-to-severe WMHs between the CSVD-n and CSVD-c groups (*P* = 0.430; *P* = 0.880; *P* = 0.493). Additionally, cognitive scale scores showed no significant differences among the three groups in the RAVLT, Logical Memory, and CDR-SB scores (*P* = 0.148; *P* = 0.197; *P* = 0.430) (Table 1). Four participants had missing RAVLT data and were excluded only from RAVLT-related analyses; no missing data were present for other variables.

**Table 1:** Demographic Characteristics of Participants

Characteristics	HC	CSVD-n	CSVD-c	P-value
n	37	78	52	-
Demographic characteristics				
Age, y, mean (SD)	68.5 (7.6)	71.4 (8.4)	71.4 (7.64)	0.144
Female, n (%)	18 (48.6%)	50 (64.1%)	29 (55.8%)	0.269
Education y, mean (SD)	16.38 (2.5)	16.05 (2.2)	16.29 (2.4)	0.743
Cognitive status, n (%)				
Cognitively normal	27 (73.0)	47 (60.3)	30 (57.7)	0.258
Mild cognitive impairment	10 (27.0)	31 (39.7)	22 (42.3)	0.258
MRI findings				
CMBs, n (%)	0 (0%)	0 (0%)	52 (100%)	< 0.001
Lacunes, n (%)	0 (0%)	43 (55.1%)	25 (48.1%)	0.430
Presence of moderate-to-severe EPVS, n (%)	0 (0%)	26 (33.3%)	18 (34.6%)	0.880
Presence of moderate-to-severe WMHs, n (%)	0 (0%)	24 (30.8%)	19 (38.5%)	0.493
Cognitive Assessments				
RAVLT, mean (SD)	0.29 (0.7)	1.4 (3.7)	1.8 (4.2)	0.148
LDELTOTAL, mean (SD)	11.7 (4.7)	11.1 (5.0)	9.9 (5.1)	0.197
CDR-SB, mean (SD)	0.4 (0.8)	0.6 (1.1)	0.6 (1.1)	0.430

Raw data were presented as mean (± standard deviation [SD]) or number (percentage, %) in tables. Continuous variables were compared with the ANOVA test, and categorical variables were compared with Pearson’s chi-squared test. Significant p values are in bold

*Abbreviations HC* Healthy controls, *CSVD* Cerebral small vessel disease, *CSVD-n* CSVD without CMBs, * CSVD-c* CSVD with CMBs, *CMBs* Cerebral microbleeds, *EPVS* Enlarged perivascular space, *WMHs* White matter hyperintensities, *RAVLT* Rey auditory verbal learning, *LDELTOTAL* Logical memory delayed recall total, *CDR-SB *Clinical dementia rating sum of the boxes

A total of 39 participants with complete baseline NODDI data and longitudinal diagnostic follow-up were included in the Cox proportional hazards regression analysis, with a mean follow-up duration of 2.74 years. During the follow-up period, 4 participants were diagnosed with incident dementia.

### Comparison of NODDI metrics among HC, CSVD-c, and CSVD-n Groups

**Fig. 1:**
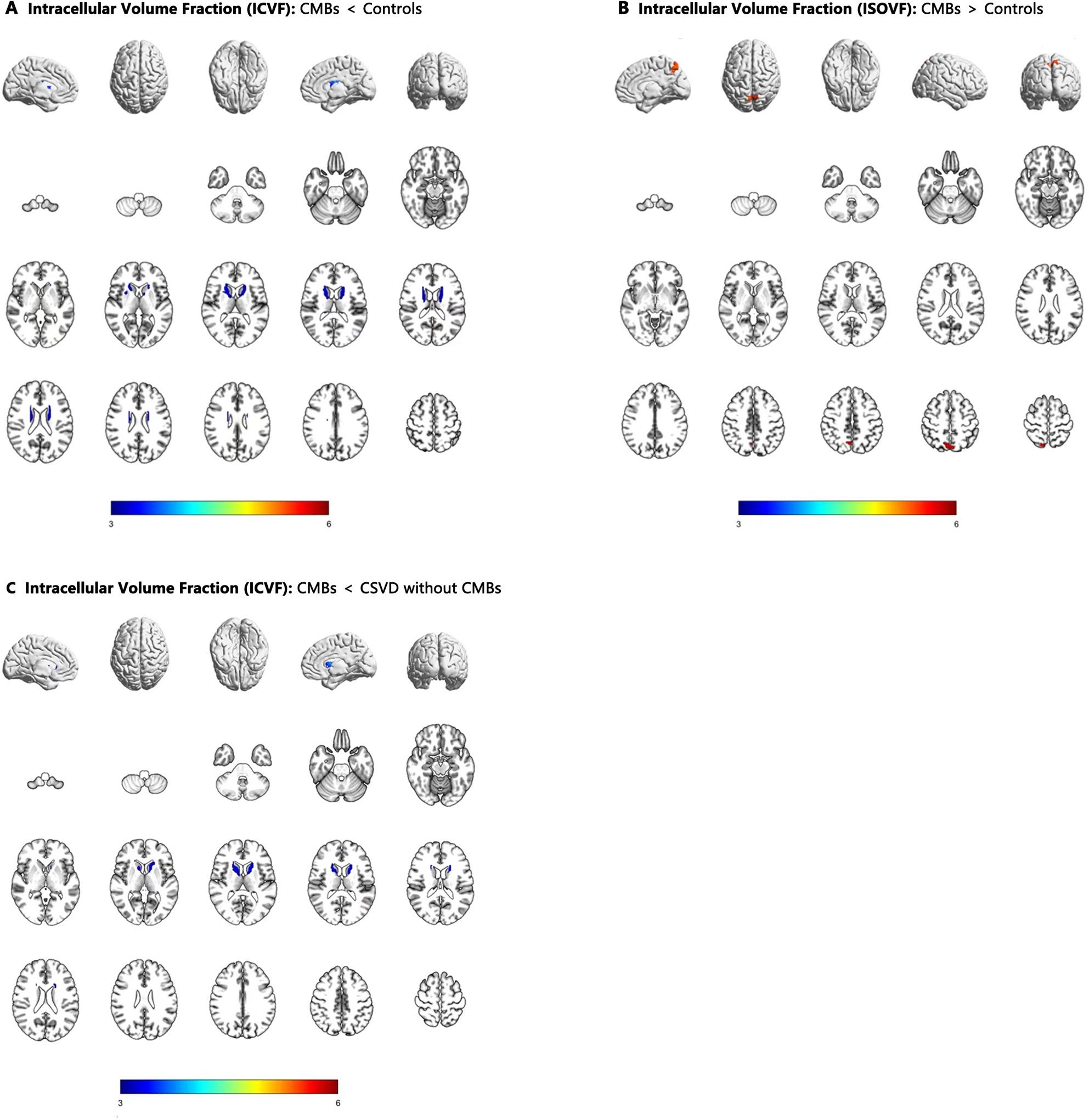
Comparison of NODDI metrics among HC, CSVD-c, and CSVD-n groups. Compared to the HC group, participants in the CSVD-c group showed decreased ICVF in the bilateral caudate nucleus (**A**) and increased ISOVF in the right precuneus (**B**). Compared to the CSVD-n group, the CSVD-c group also showed decreased ICVF in the bilateral caudate nucleus with relatively smaller scope (**C**). Blue and red clusters denote regions with significantly decreased and increased NODDI metrics, respectively (*P* < 0.05, FDR corrected). ICVF, Intracellular Volume Fraction; ISOVF, Isotropic Volume Fraction; HC, Healthy controls; CSVD-c, Cerebral small vessel disease patients with cerebral microbleeds; CSVD-n, Cerebral small vessel disease patients without cerebral microbleeds

Compared to the HC group, participants in the CSVD-c group showed decreased ICVF in the bilateral caudate nucleus and increased ISOVF in the right precuneus. There was no significant difference in ODI between the CSVD-c group and the control group. Similarly, compared to the CSVD-n group, the CSVD-c group showed decreased ICVF in the bilateral caudate nucleus with relatively smaller scope (FDR corrected, *P* < 0.05, Fig. 1). However, there was no significant difference between the CSVD-n and CSVD-c groups in ISOVF and ODI. The cluster size, peak coordinates, and T statistics of the voxel-wise comparisons of NODDI metrics among the three groups are presented in Table 2. Results were consistent after further adjustment for cognitive diagnosis (CN/MCI), as detailed in Supplementary Table 1.

**Table 2:** Cerebral Clusters with Significantly Altered Diffusion Metrics (ICVF and ISOVF) between CSVD-c and Control Groups, and CSVD-c and CSVD-n Groups (P < 0.05, FDR Corrected)

NODDI metrics	Cluster size(voxels)	Peak voxel	Regions
		MNI Coordinates (mm)	
		x y z	
ICVF (CSVD-c < HC)	438	22 20 12	Right caudate
	381	-20 20 14	Left caudate
ISOVF (CSVD-c > HC)	264	10 -62 48	Right precuneus
ICVF (CSVD-c < CSVD-n)	288	-14 10 12	Left caudate
	213	12 8 8	Right caudate

Anatomical localizations of peak MNI coordinates were established according to the cortical Desikan atlas [48] and subcortical FreeSurfer ASEG atlases

*Abbreviations ICVF* Intracellular Volume Fraction, *ISOVF* Isotropic Volume Fraction, *CSVD* Cerebral small vessel disease, *CSVD-n* CSVD without CMBs, *CSVD-c* CSVD with CMBs, * CMBs * Cerebral microbleeds, * HC* Healthy controls, * FDR* False discovery rate

### Correlation with CMBs

**Fig. 2:**
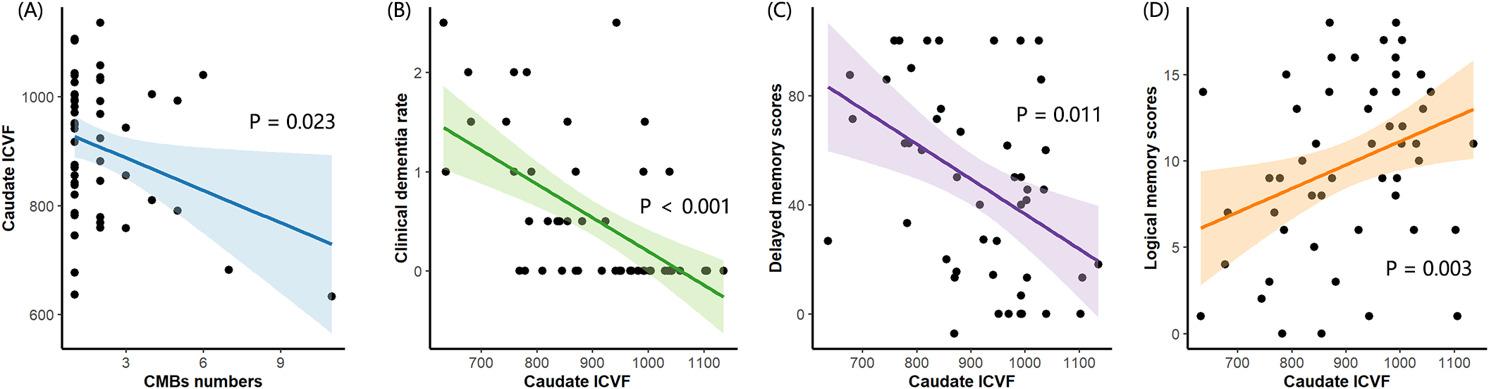
Correlations between ICVF in the caudate nucleus and CMBs count (**A**), CDR-SB scores (**B**), delayed memory scores (**C**), and logical memory scores (**D**). Reduced ICVF in the bilateral caudate nucleus is significantly associated with increased CMBs count, higher CDR-SB scores, and impaired delayed and logical memory. ICVF values were multiplied by 1000 for better visualization in scatter plots. ICVF, Intracellular Volume Fraction; CMBs, Cerebral microbleeds; CDR-SB, Clinical Dementia Rating Sum of Boxes

Decreased ICVF value in the bilateral caudate nucleus was significantly correlated with the number of CMBs (*P* = 0.023, β = -0.40). No significant correlation was found between increased ISOVF in the right precuneus and CMBs (*P* = 0.329, β = -0.14) (Fig. 2).

### Correlations with neuropsychological measurements

Multiple linear regression analysis, adjusted for age, sex, and years of education, revealed that decreased ICVF values in the bilateral caudate nucleus were significantly correlated with lower Logical Memory scores (*P* = 0.003, β = 0.47) and higher RAVLT and CDR-SB scores (*P* = 0.011, β = -0.45; *P* < 0.001, β = -0.62) (Fig. 2). Other MRI markers, including the number of CMBs (Logical Memory: *P* = 0.387, β = -0.12; RAVLT: *P* = 0.337, β = 0.14; CDR-SB: *P* = 0.363, β = 0.12), the location of CMBs (Logical Memory: *P* = 0.220, β = -0.16; RAVLT: *P* = 0.318, β = 0.14; CDR-SB: *P* = 0.765, β = -0.04), the total burden of CSVD (Logical Memory: *P* = 0.171, β = -0.20; RAVLT: *P* = 0.242, β = 0.19; CDR-SB: *P* = 0.252, β = 0.17), and the increased ISOVF value in the right precuneus (Logical Memory: *P* = 0.455, β = 0.100; RAVLT: *P* = 0.382, β = -0.127; CDR-SB: *P* = 0.804, β = -0.034), were not correlated with Logical Memory, CDR-SB, or RAVLT scores.

When the additional covariate TIV was included, the results were consistent with those in Model 1. Decreased ICVF values in the bilateral caudate nucleus remained significantly correlated with lower Logical Memory scores (*P* = 0.007, β = 0.44) and higher RAVLT and CDR-SB scores (*P* = 0.011, β = -0.46; *P* < 0.001, β = -0.57) (Fig. 2). Other MRI markers, including the number of CMBs (Logical Memory: *P* = 0.573, β = -0.08; RAVLT: *P* = 0.342, β = 0.14; CDR-SB: *P* = 0.072, β = 0.24), the location of CMBs (Logical Memory: *P* = 0.345, β = -0.13; RAVLT: *P* = 0.295, β = 0.16; CDR-SB: *P* = 0.923, β = -0.01), the total burden of CSVD (Logical Memory: *P* = 0.178, β = -0.19; RAVLT: *P* = 0.248, β = 0.19; CDR-SB: *P* = 0.061, β = 0.26), and the increased ISOVF value in the right precuneus (Logical Memory: *P* = 0.455, β = 0.100; RAVLT: *P* = 0.382, β = -0.127; CDR-SB: *P* = 0.804, β = -0.034), were not significantly correlated with Logical Memory, CDR-SB, or RAVLT scores. Table 3 summarizes the detailed results of these correlations between NODDI metrics, CMBs characteristics, and cognitive performance across both regression models.

**Table 3:** Correlations Between NODDI metric, CMBs Characteristics, and Cognitive Performance

	Model1		Model2	
	β	*P* value	β	*P* value
Logical Memory				
Caudate ICVF	0.47	0.003	0.44	0.007
CMBs count	-0.12	0.387	-0.08	0.573
CMBs location	-0.16	0.220	-0.13	0.345
CSVD burden	-0.20	0.171	-0.19	0.178
RAVLT				
Caudate ICVF	-0.45	0.011	-0.46	0.011
CMBs count	0.14	0.337	0.14	0.342
CMBs location	0.14	0.318	0.16	0.295
CSVD burden	0.19	0.242	0.19	0.248
CDR-SB				
Caudate ICVF	-0.62	< 0.001	-0.57	< 0.001
Number of CMBs	0.12	0.363	0.24	0.072
Location of CMBs	-0.04	0.765	-0.01	0.923
CSVD burden	0.17	0.252	0.26	0.061

Multiple regression analysis was conducted. Model 1 was adjusted for age, sex, and years of education, while Model 2 included an additional adjustment for TIV. P-values that reached statistical significance are highlighted in bold. No multiple comparison correction was applied for these correlation analyses to avoid overly conservative inferences due to the relatively small sample size

*Abbreviations NODDI* Neurite Orientation Dispersion and Density Imaging, *CSVD* Cerebral Small Vessel Disease, *ICVF * Intracellular Volume Fraction, *CMBs *Cerebral Microbleeds, *TIV* Total intracranial volume, *CDR-SB* Clinical Dementia Rating Sum-of-Boxes

### ISOVF in right precuneus and dementia risk

In the univariate Cox regression analysis, we found that increased ISOVF in the right precuneus was significantly associated with the risk of dementia (HR = 1.016, 95% CI = 1.001–1.031, *P* = 0.039). After adjusting for potential confounders of sex, age, and education level, the association between ISOVF in the right precuneus and the risk of the event was attenuated and became non-significant, remaining a trend towards significance (HR = 1.025, 95% CI = 0.989–1.062, *P* = 0.182).

## Discussion

In this study, we observed a significant decrease in the ICVF value of the bilateral caudate nucleus and an increase in the ISOVF value of the right precuneus in CSVD patients with CMBs compared to both the HC group and CSVD patients without CMBs. The reduction in ICVF in the bilateral caudate nucleus was closely correlated with episodic memory, delayed memory, and overall cognitive function. The increased ISOVF in the right precuneus was significantly associated with the risk of dementia. Additionally, our findings suggest that the ICVF of the bilateral caudate is more strongly correlated with cognitive function than traditional MRI markers.

Few previous studies have focused mainly on CMBs, concentrating instead on overall CSVD. A study on hypertensive patients using enhanced susceptibility weighted angiography found reduced phase and magnitude values in the head of the caudate nucleus in those with CMBs, correlating with disease duration and blood pressure [19]. Mengmeng Feng et al. reported that patients with severe CSVD showed significantly decreased nodal efficiency in the bilateral caudate nucleus, which was negatively correlated with all cognitive parameters [20]. Similarly, Yachen Shi et al. found that impaired amplitude of low-frequency fluctuation values in the bilateral caudate of cognitively impaired CSVD patients significantly correlated with global cognitive function, executive function, and information processing speed [21]. Xinyue Zhang et al. identified significantly decreased amplitude of low-frequency fluctuation/voxel-based morphometry coupling in the bilateral caudate nuclei in moderate and severe CSVD groups compared to healthy controls, with decoupling values correlating with attention and executive functions, distinguishing between CSVD subtypes [22]. These findings align with our study, indicating the caudate nucleus plays a critical role in microbleed-related cognitive impairment.

By directly comparing CSVD patients with and without CMBs, our study uniquely identified that CMBs are independently associated with reduced ICVF in the bilateral caudate nucleus, above and beyond other CSVD markers. This microstructural alteration was directly correlated with CMB count and cognitive function, which cannot be explained by conventional CSVD burden alone. These findings clarify that CMBs exert an independent, deleterious effect on neurite density that is separable from other small vessel changes, advancing mechanistic understanding of CMB‑related cognitive decline. This significant correlation between bilateral caudate ICVF and cognitive function was identified in the CSVD-c group, where our analysis focused given the study’s core aim to explore CMB-related cognitive impairment mechanisms. This finding uncovers a CMB-specific association between microstructural injury and cognitive function undetectable by intergroup cognitive score comparisons, highlighting NODDI’s superior sensitivity in capturing such early subclinical changes in CMB patients.

The caudate nucleus, a principal component of the corpus striatum and part of the dorsal striatum along with the putamen, is a major input pathway of the basal ganglia [23]. Numerous neuroimaging studies have linked the caudate nucleus to cognitive functions, especially memory. As early as 1956, Rosvold et al. reported that electrical stimulation of the caudate nucleus impaired declarative memory [24]. Subsequent case reports, including that of De Coteau et al. [25], suggested that the damage of the medial caudate nucleus led to short-term memory of orientation information. Functional imaging studies in both primates and humans have shown activation of the subcortical loop during working memory tasks, suggesting a role for the caudate nucleus in working memory deficits before disease onset [26]. Structural imaging studies have found that the volume of the caudate nucleus negatively correlates with persistent errors in spatial working memory tasks. In our study, patients with CMBs exhibited significantly decreased ICVF in the bilateral caudate, which was significantly correlated with overall cognitive function, episodic memory, and delayed memory [27]. This suggests that intracerebral microbleeds may indirectly affect neuron density (measured by ICVF), ultimately leading to cognitive impairment.

Furthermore, we found that the increased ISOVF in the right precuneus was significantly associated with the risk of dementia. However, the prognostic effect size of this metric was small, and the association only trended toward significance after adjustment for potential confounders including age, sex, and years of education. Since ISOVF is a normalized metric ranging from 0 to 1 and shows only subtle percentage changes between groups, the corresponding increase in dementia risk is modest. ISOVF may be regarded as a surrogate marker of early microstructural injury rather than a robust predictive tool for individualized dementia prognosis. This link between ISOVF and dementia risk should be regarded as an exploratory finding, requiring further validation in larger cohorts.

Additionally, we compared the NODDI metrics with traditional MRI markers, including the number and location of CMBs, and the total CSVD burden. Our findings indicate that NODDI metrics were most strongly correlated with cognitive function in patients with CMBs. The multi-shell diffusion imaging model, NODDI, differentiates the DWI signal into three distinct compartments: intraneuronal (within neurites), extraneuronal (outside neurites), and cerebrospinal fluid (free water). This technique has significantly enhanced our ability to study and characterize cortical microstructure in detail. NODDI has been employed in various central nervous system diseases, including multiple sclerosis, Parkinson’s disease, and Alzheimer’s disease, demonstrating its value as an imaging tool. For instance, multiple studies assessed NODDI metrics of gray matter in multiple sclerosis and controls. These studies found that ICVF of multiple cortical lesions was decreased compared to the normal-appearing gray matter of patients and to the normal gray matter tissue from HCs [16]. Nicholas M. Vogt et al. report that, for participants with MCI, gray matter ICVF, but not cortical thickness, was decreased in temporal, parietal, and posterior cingulate regions, highlighting the utility of NODDI metrics in detecting cortical microstructural degeneration that occurs prior to measurable macrostructural changes and overt clinical dementia [28]. However, the added benefits of multi-shell diffusion imaging and advanced diffusion modeling in CMBs remain largely unexplored. The current study suggests that NODDI is a promising technique for detecting imaging markers of cognitive impairment and the risk of dementia in patients with CMBs. Future longitudinal studies are needed to confirm these findings.

We acknowledge several limitations in this study. Firstly, the sample size is relatively small, necessitating validation of our findings in a larger cohort. In addition, only four incident dementia cases were included in the Cox regression model, which may compromise the stability of hazard ratio estimates and reduce statistical power. We also acknowledge that no multiple comparison correction was performed for the correlation analyses, a compromise to avoid overly conservative inferences given the relatively small sample size. Secondly, patients with only CMBs were not included, limiting our ability to isolate the effects of CMBs on brain structure and function. However, it is noteworthy that in real-world scenarios, individuals presenting with only CMBs are exceedingly rare. To address this limitation, we included two control groups: one comprising participants with a CSVD burden score of 0, and another with CSVD characteristics but without microbleeds. By comparing these groups, we aim to elucidate the impact of CMBs on the brain, thus providing valuable insights despite the constraints.

In conclusion, our study found that patients with CMBs exhibit decreased ICVF in the bilateral caudate nucleus and increased ISOVF in the right precuneus, with ICVF closely associated with cognitive impairment and ISOVF showing an exploratory association with dementia risk. NODDI metrics may outperform traditional neuroimaging markers, serving as valuable markers for investigating CMB-related cognitive impairment.

## Materials and methods

### Study participant recruitment

Data used in the preparation of this article were obtained from the Alzheimer's Disease Neuroimaging Initiative database (available at https://adni.loni.usc.edu). 78 CSVD patients without CMBs (CSVD-n), 52 CSVD patients with CMBs (CSVD-c), and 37 age-, sex-, and education year-matched healthy subjects with baseline NODDI scan were recruited. Inclusion criteria for CSVD were based on the Standards for Reporting Vascular changes on Neuroimaging [1]. Exclusion criteria included a history of neurologic or psychiatric disorders such as Parkinson’s disease, multi-infarct dementia, Huntington’s disease, normal pressure hydrocephalus, brain tumor, progressive supranuclear palsy, seizure disorder, subdural hematoma, multiple sclerosis, or history of significant head trauma followed by persistent neurologic deficits or known structural brain abnormalities. For the longitudinal analysis of incident dementia risk, participants were further included if they had completed at least the second follow-up visit, with available longitudinal cognitive diagnostic information and complete baseline NODDI imaging data. Dementia was diagnosed by a multidisciplinary clinical panel incorporating the NINCDS-ADRDA criteria for Alzheimer’s dementia and the DSM‑IV diagnostic criteria for dementia, with comprehensive assessment of cognitive performance, functional status and neuroimaging findings.

### Evaluation of CSVD imaging markers

CSVD imaging markers were evaluated according to the Standards for Reporting Vascular Changes on Neuroimaging criteria [1, 29] by two experienced neurologists.

### CMBs

CMBs were identified as uniform hypointense lesions of up to 10 mm in diameter on T2*-weighted MRI images [30]. The identification of the number and locations of CMBs was performed by trained imaging analysts and verified by radiologists specialized in T2* imagery. CMBs were recorded as ‘present’ if at least one CMB was visible, and ‘absent’ if no CMBs were detected. Furthermore, the locations of CMBs were categorized into lobar CMBs, deep (basal ganglia) CMBs, cerebellar/brainstem CMBs, or mixed CMBs regions.

### WMHs

WMHs were quantified using both the Fazekas scale and white matter hyperintensity volume (WMHV). The WMHV was automatically segmented utilizing a deep-learning method refined on the United Imaging platform [31]. The Fazekas scale, applied by two proficient neurologists, separately assessed periventricular and deep WMHs [32]. Periventricular WMHs with scores greater than or equal to 2 or deep WMHs with scores greater than or equal to 3 were defined as having moderate to severe WMHs.

### Lacunes

Lacunes were defined as cerebral spinal fluid-like hypointensities, 3 to 15 mm in diameter, encircled by a hyperintense rim on T2 FLAIR images [33]. The distinction from EPVS was based on size (larger than 2 mm and up to 15 mm) and the characteristic hyperintense rim on FLAIR.

### EPVS

EPVS were assessed and categorized within the basal ganglia. The count was conducted on both hemispheres on the MRI slice exhibiting the maximal extent of EPVS, with scoring as follows: 0 = no EPVS; 1 = 1 to 10 EPVS; 2 = 11 to 20 EPVS; 3 = 21 to 40 EPVS; and 4 = more than 40 EPVS. EPVS scores of 3 to 4 were categorized as moderate to severe levels.

### CSVD burden

The overall burden of CSVD was determined by assigning one point for each of the following: a periventricular Fazekas score of 3 or a deep Fazekas score of 2 or higher, the presence of one or more lacunes, the presence of one or more microbleeds, and a BG-EPVS grade of 2 or 3. Consequently, the total CSVD score ranged from 0 to 4.

### MRI data acquisition

#### 3D T1-weighted image acquisition

All participants underwent MRI scanning on a 3.0-T MRI system (Siemens). Three-dimensional T1-weighted magnetization prepared rapid acquisition with gradient echo images were obtained as follows: Flip Angle=9.0 degree; Matrix X=240.0 pixels; Matrix Y=256.0 pixels; Matrix Z=208.0; Pixel Spacing X=1.0 mm; Pixel Spacing Y=1.0 mm; Pulse Sequence=GR/IR; Slice Thickness=1.0 mm; TE=3.0 ms; TI=900.0 ms; TR=2300.0 ms;

#### Diffusion image acquisition

For the diffusion imaging, a multi-shell protocol was acquired along 112 total diffusion weighted directions at three b-values (500,1000, 2000 s/mm2). The diffusion MRI parameters were as follows: Flip Angle=90.0 degree; Matrix X=240.0 pixels; Matrix Y=256.0 pixels; Pixel Size X=2.0 mm; Pixel Size Y=2.0 mm; Pulse Sequence=EP; Slice Thickness=2.0 mm; TE=71.0 ms; TR=3400.0 ms;

### MRI data processing

#### Voxel-based morphometry analysis

The Computational Anatomy Toolbox (CAT12) was used for voxel-based morphometry of the imaging data. Default parameter settings were used including realignment, bias correction, tissue classification, and spatial normalization using the DARTEL template. Images were segmented into gray matter, white matter, and cerebrospinal fluid. Next, the images were transformed to the Montreal Neurological Institute (MNI) standard space. Finally, the normalized, modulated gray matter segmentations were smoothed with a 6-mm full-width at half maximum Gaussian kernel to improve the signal-to-noise ratio. The total intracranial volume (TIV) was estimated by summing the volumes of gray matter, white matter and cerebrospinal fluid (WMHs are automatically included in white matter volume segmentation and thus in TIV calculation, as they form part of intracranial brain parenchyma).

#### NODDI analysis [34, 35]

For each subject’s data, the quantitative metrics from NODDI model (ICVF, ODI, and ISOVF) were calculated using an in-house developed tool called NeuDiLab, which was based on Python 3.5 and open-resource projects previously validated [36–38]. The processing pipeline included the following steps: 1) the skull was removed by BET from FSL [39]; 2) eddy current distortion and motion artifacts were corrected using the bneddy tool from DiffusionKit [40]; 3) a 3D Gaussian filter was applied to diffusion data with a full width at half maximum Full Width at Half Maximum of 3 mm to increase SNR and reduce potential misregistration among diffusion data. The Full Width at Half Maximum was set at 1.5 times of voxel size according to previous studies[41, 42]; 4) the NODDI model metrics were estimated by algorithms from AMICO [43]. The normalization process included the following steps: 1) b0 image was co-registered to the T1 image; 2) T1 image was spatially normalized into the MNI space; 3) Based on the b0-T1 and T1-MNI registration files, the DWI metrics were transformed into the MNI space; 4) The normalized DWI metrics were further spatially smoothed with a 6-mm Full Width at Half Maximum Gaussian kernel. All these analyses were performed using SPM12.

### Neuropsychological assessments

For correlation analysis between imaging and cognition, we used Clinical Dementia Rating Sum-of-Boxes (CDR-SB) as a measure of global cognitive function [44], the Wechsler Memory Scale-revised Logical Memory II for delayed recall [45], the Rey Auditory Verbal Learning Test (RAVLT) for episodic memory [46].

CDR-SB is a refined version of the original CDR scale developed by Berg in 1988 [47], offering a more detailed assessment of dementia severity. The CDR evaluates six domains: memory, orientation, judgment and problem-solving, community affairs, home and hobbies, and personal care. Unlike the Global CDR score, which provides an overall rating, the CDR-SB delivers a precise evaluation by summing the individual scores from each domain. Both the CDR-SB and Global CDR scores are automatically calculated within the electronic Case Report Form.

The RAVLT test is a list learning task which assesses learning and memory. On each of 5 learning trials, 15 unrelated nouns are presented orally at the rate of 1 word per second and immediate free recall of the words is elicited. After a 30-minute delay filled with unrelated testing, free recall of the original 15-word list is elicited. Both immediate recall and the percent forgotten are used [46].

The Logical Memory tests (Delayed Paragraph Recall) is from the Wechsler Memory Scale–Revised. Free recall of 1 short story is elicited immediately after being read aloud to the participant and again after a 30-minute delay. The total bits of information recalled after the delay interval (maximum score = 25) are analyzed [45].

### Statistics

Statistical analyses were conducted using R version 4.2.1, with a two-tailed P value < 0.05 considered statistically significant. One-way analysis of variance was used for intergroup comparisons of continuous baseline demographic and cognitive variables, while the Pearson’s chi-square test was applied for dichotomous variables. Missing data were handled by complete-case analysis.

For NODDI metric analyses, key differential brain regions were first identified via voxel-wise intergroup comparisons using SPM12 (https://www.fil.ion.ucl.ac.uk/spm/), with the general linear model implemented at a statistical threshold of uncorrected P < 0.001 and FDR-corrected P < 0.05. Covariates for this voxel-based analysis included age, sex, years of education and cognitive diagnosis, and the analysis was restricted to a gray matter mask defined by SPM12 built-in tissue probability maps (gray matter probability threshold ≥10%). Mean NODDI metric values of these identified differential regions were then extracted from the FreeSurfer Desikan-Killiany anatomical atlas for subsequent statistical analyses, and all voxel-wise analysis results were visualized using BrainNet Viewer (http://www.nitrc.org/projects/bnv/).

Multiple linear regression analysis restricted to the CSVD-c group was performed to examine the associations between cognitive scores and NODDI metrics, with age, sex, years of education and cognitive diagnosis as covariates in Model 1; total intracranial volume (TIV) was additionally included as a covariate in Model 2. Univariate and multivariable Cox proportional hazards regression analyses were conducted to assess the relationship between right precuneus ISOVF values and incident dementia risk, where the multivariable model was adjusted for age, sex, and years of education.

## Supplementary Information

Supplementary Material 1.

## Data Availability

Data used in this study are available through the ADNI database ( [https://ida.loni.usc.edu/](https:/ida.loni.usc.edu) ). Data available on request from the authors.

## Abbreviations

CMBs: Cerebral Microbleeds
CSVD: Cerebral Small Vessel Disease
WMHs: White Matter Hyperintensities
EPVSs: Enlarged Perivascular Spaces
NODDI: Neurite Orientation Dispersion and Density Imaging
ICVF: Intracellular Volume Fraction
ODI: Orientation Dispersion Index
ISOVF: Isotropic Volume Fraction
CDR-SB: Clinical Dementia Rating Sum-of-Boxes
RAVLT: Rey Auditory Verbal Learning Test
CSVD-c: CSVD Patients with CMBs
CSVD-n: CSVD Patients without CMBs
HC: Healthy Controls
DTI: Diffusion Tensor Imaging
TIV: Total Intracranial Volume

## References

[1] Duering M, Biessels GJ, Brodtmann A, Chen C, Cordonnier C, de Leeuw FE, Debette S, Frayne R, Jouvent E, Rost NS, et al. Neuroimaging standards for research into small vessel disease-advances since 2013. Lancet Neurol. 2023;22(7):602–18. 37236211 10.1016/S1474-4422(23)00131-X

[2] Agarwal A, Ajmera P, Sharma P, Kanekar S. Cerebral microbleeds: Causes, clinical relevance, and imaging approach - A narrative review. J neurosciences rural Pract. 2024;15(2):169–81. 10.25259/JNRP_351_2023 PMC11090589 38746527

[3] Charidimou A, Smith EE. Cardiovascular Management in Asymptomatic (Silent) Cerebral Microbleeds and Suspected Cerebral Amyloid Angiopathy. Stroke. 2024;55(4):1101–12. 38465605 10.1161/STROKEAHA.123.044167

[4] Csiszar A, Ungvari A, Patai R, Gulej R, Yabluchanskiy A, Benyo Z, Kovacs I, Sotonyi P, Kirkpartrick AC, Prodan CI et al. Atherosclerotic burden and cerebral small vessel disease: exploring the link through microvascular aging and cerebral microhemorrhages. *GeroScience* 2024. 10.1007/s11357-024-01139-7 PMC11336042 38639833

[5] Wilson D, Ambler G, Lee KJ, Lim JS, Shiozawa M, Koga M, Li L, Lovelock C, Chabriat H, Hennerici M, et al. Cerebral microbleeds and stroke risk after ischaemic stroke or transient ischaemic attack: a pooled analysis of individual patient data from cohort studies. Lancet Neurol. 2019;18(7):653–65. 31130428 10.1016/S1474-4422(19)30197-8 PMC6562236

[6] Wilson D, Ambler G, Shakeshaft C, Brown MM, Charidimou A, Al-Shahi Salman R, Lip GYH, Cohen H, Banerjee G, Houlden H, et al. Cerebral microbleeds and intracranial haemorrhage risk in patients anticoagulated for atrial fibrillation after acute ischaemic stroke or transient ischaemic attack (CROMIS-2): a multicentre observational cohort study. Lancet Neurol. 2018;17(6):539–47. 29778365 10.1016/S1474-4422(18)30145-5 PMC5956310

[7] Cordonnier C, van der Flier WM, Sluimer JD, Leys D, Barkhof F, Scheltens P. Prevalence and severity of microbleeds in a memory clinic setting. Neurology. 2006;66(9):1356–60. 16682667 10.1212/01.wnl.0000210535.20297.ae

[8] Shams S, Martola J, Granberg T, Li X, Shams M, Fereshtehnejad SM, Cavallin L, Aspelin P, Kristoffersen-Wiberg M, Wahlund LO. Cerebral microbleeds: different prevalence, topography, and risk factors depending on dementia diagnosis—the Karolinska Imaging Dementia Study. AJNR Am J Neuroradiol. 2015;36(4):661–6. 25523590 10.3174/ajnr.A4176 PMC7964321

[9] Nikseresht G, Evia AM, Nag S, Leurgans SE, Capuano AW, Agam G, Barnes LL, Bennett DA, Schneider JA, Arfanakis K. Neuropathologic correlates of cerebral microbleeds in community-based older adults. Neurobiol Aging. 2023;129:89–98. 37279617 10.1016/j.neurobiolaging.2023.05.005 PMC10524842

[10] Boyle PA, Yu L, Nag S, Leurgans S, Wilson RS, Bennett DA, Schneider JA. Cerebral amyloid angiopathy and cognitive outcomes in community-based older persons. Neurology. 2015;85(22):1930–6. 26537052 10.1212/WNL.0000000000002175 PMC4664125

[11] Sepehry AA, Rauscher A, Hsiung GY, Lang DJ. Microbleeds in Alzheimer’s Disease: A Neuropsychological Overview and Meta-Analysis. Can J Neurol Sci Le J canadien des Sci neurologiques. 2016;43(6):753–9. 10.1017/cjn.2016.296 27640605

[12] Beaman C, Kozii K, Hilal S, Liu M, Spagnolo-Allende AJ, Polanco-Serra G, Chen C, Cheng CY, Zambrano D, Arikan B, et al. Cerebral Microbleeds, Cerebral Amyloid Angiopathy, and Their Relationships to Quantitative Markers of Neurodegeneration. Neurology. 2022;98(16):e1605–16. 35228332 10.1212/WNL.0000000000200142 PMC9052569

[13] Sui C, Wen H, Wang S, Feng M, Xin H, Gao Y, Li J, Guo L, Liang C. Characterization of white matter microstructural abnormalities associated with cognitive dysfunction in cerebral small vessel disease with cerebral microbleeds. J Affect Disord. 2023;324:259–69. 36584708 10.1016/j.jad.2022.12.070

[14] Andica C, Kamagata K, Hatano T, Saito Y, Ogaki K, Hattori N, Aoki S. MR Biomarkers of Degenerative Brain Disorders Derived From Diffusion Imaging. J Magn Reson imaging: JMRI. 2020;52(6):1620–36. 31837086 10.1002/jmri.27019 PMC7754336

[15] Kamiya K, Hori M, Aoki S. NODDI in clinical research. J Neurosci Methods. 2020;346:108908. 32814118 10.1016/j.jneumeth.2020.108908

[16] Seyedmirzaei H, Nabizadeh F, Aarabi MH, Pini L. Neurite Orientation Dispersion and Density Imaging in Multiple Sclerosis: A Systematic Review. J Magn Reson imaging: JMRI. 2023;58(4):1011–29. 37042392 10.1002/jmri.28727

[17] Wei X, Wang S, Zhang M, Yan Y, Wang Z, Wei W, Tuo H, Wang Z. Gait impairment-related axonal degeneration in Parkinson’s disease by neurite orientation dispersion and density imaging. NPJ Parkinson’s disease. 2024;10(1):45. 38413647 10.1038/s41531-024-00654-w PMC10899173

[18] Mak E, Reid RI, Przybelski SA, Lesnick TG, Schwarz CG, Senjem ML, Raghavan S, Vemuri P, Jack CR Jr., Min HK, et al. Influences of amyloid-β and tau on white matter neurite alterations in dementia with Lewy bodies. NPJ Parkinson’s disease. 2024;10(1):76. 38570511 10.1038/s41531-024-00684-4 PMC10991290

[19] Yang J, Yang Z, Wu H, Chen W. Quantification of Iron Deposition in the Brain of Hypertensive Patients Using 3D-enhanced Susceptibility-weighted Angiography (ESWAN). *Current medical imaging* 2023. 10.2174/1573405620666230627112146 37366358

[20] Feng M, Wen H, Xin H, Wang S, Gao Y, Sui C, Liang C, Guo L. Decreased Local Specialization of Brain Structural Networks Associated with Cognitive Dysfuntion Revealed by Probabilistic Diffusion Tractography for Different Cerebral Small Vessel Disease Burdens. Mol Neurobiol. 2024;61(1):326–39. 37606718 10.1007/s12035-023-03597-0 PMC10791730

[21] Shi Y, Mao H, Miao W, Deng J, Gao Q, Zeng S, Ma L, Han Y, Ji W, Li Y, et al. Potential Association of Neutrophil Extracellular Traps With Cognitive Impairment in Cerebral Small Vessel Disease. journals Gerontol Ser Biol Sci Med Sci. 2023;78(11):1999–2006. 10.1093/gerona/glad184 37527839

[22] Zhang X, Liang C, Wang N, Wang Y, Gao Y, Sui C, Xin H, Feng M, Guo L, Wen H. Abnormal whole-brain voxelwise structure-function coupling and its association with cognitive dysfunction in patients with different cerebral small vessel disease burdens. Front Aging Neurosci. 2023;15:1148738. 37455935 10.3389/fnagi.2023.1148738 PMC10347527

[23] Yager LM, Garcia AF, Wunsch AM, Ferguson SM. The ins and outs of the striatum: role in drug addiction. Neuroscience. 2015;301:529–41. 26116518 10.1016/j.neuroscience.2015.06.033 PMC4523218

[24] Rosvold HE, Delgado JM. The effect on delayed-alternation test performance of stimulating or destroying electrically structures within the frontal lobes of the monkey’s brain. J Comp physiological Psychol. 1956;49(4):365–72. 10.1037/h0087991 13345915

[25] DeCoteau WE, Hoang L, Huff L, Stone A, Kesner RP. Effects of hippocampus and medial caudate nucleus lesions on memory for direction information in rats. Behav Neurosci. 2004;118(3):540–5. 15174931 10.1037/0735-7044.118.3.540

[26] Hannan KL, Wood SJ, Yung AR, Velakoulis D, Phillips LJ, Soulsby B, Berger G, McGorry PD, Pantelis C. Caudate nucleus volume in individuals at ultra-high risk of psychosis: a cross-sectional magnetic resonance imaging study. Psychiatry Res. 2010;182(3):223–30. 20488675 10.1016/j.pscychresns.2010.02.006

[27] Levitt JJ, McCarley RW, Dickey CC, Voglmaier MM, Niznikiewicz MA, Seidman LJ, Hirayasu Y, Ciszewski AA, Kikinis R, Jolesz FA, et al. MRI study of caudate nucleus volume and its cognitive correlates in neuroleptic-naive patients with schizotypal personality disorder. Am J Psychiatry. 2002;159(7):1190–7. 12091198 10.1176/appi.ajp.159.7.1190 PMC2826363

[28] Vogt NM, Hunt JF, Adluru N, Dean DC, Johnson SC, Asthana S, Yu JJ, Alexander AL, Bendlin BB. Cortical Microstructural Alterations in Mild Cognitive Impairment and Alzheimer’s Disease Dementia. *Cerebral cortex (New York, NY*: 1991) 2020, 30(5):2948–2960. 10.1093/cercor/bhz286 PMC7197091 31833550

[29] Wardlaw JM, Smith EE, Biessels GJ, Cordonnier C, Fazekas F, Frayne R, Lindley RI, O’Brien JT, Barkhof F, Benavente OR, et al. Neuroimaging standards for research into small vessel disease and its contribution to ageing and neurodegeneration. Lancet Neurol. 2013;12(8):822–38. 23867200 10.1016/S1474-4422(13)70124-8 PMC3714437

[30] Lyall DM, Muñoz Maniega S, Harris SE, Bastin ME, Murray C, Lutz MW, Saunders AM, Roses AD, Valdés Hernández Mdel C, Royle NA, et al. APOE/TOMM40 genetic loci, white matter hyperintensities, and cerebral microbleeds. Int J stroke: official J Int Stroke Soc. 2015;10(8):1297–300. 10.1111/ijs.12615 PMC4950052 26310205

[31] Liu S, Jie C, Zheng W, Cui J, Wang Z. Investigation of Underlying Association Between Whole Brain Regions and Alzheimer’s Disease: A Research Based on an Artificial Intelligence Model. Front Aging Neurosci. 2022;14:872530. 35747447 10.3389/fnagi.2022.872530 PMC9211045

[32] Patti J, Helenius J, Puri AS, Henninger N. White Matter Hyperintensity-Adjusted Critical Infarct Thresholds to Predict a Favorable 90-Day Outcome. Stroke. 2016;47(10):2526–33. 27633020 10.1161/STROKEAHA.116.013982 PMC5039097

[33] Luo X, Jiaerken Y, Yu X, Huang P, Qiu T, Jia Y, Li K, Xu X, Shen Z, Guan X, et al. Associations between APOE genotype and cerebral small-vessel disease: a longitudinal study. Oncotarget. 2017;8(27):44477–89. 28574812 10.18632/oncotarget.17724 PMC5546495

[34] Huang W, Dong X, Zhao T, Kucikova L, Fu A, Shu N. DCP: A pipeline toolbox for diffusion connectome. Hum Brain Mapp. 2024;45(3):e26626. 38375916 10.1002/hbm.26626 PMC10877999

[35] Zhang J, Hu q, Li F, Wu G. Cortical microstructural alterations along the Alzheimer’s disease continuum and association with amyloid and tau pathology; 2024.

[36] Chen HJ, Zhan C, Cai LM, Lin JH, Zhou MX, Zou ZY, Yao XF, Lin YJ. White matter microstructural impairments in amyotrophic lateral sclerosis: A mean apparent propagator MRI study. NeuroImage Clin. 2021;32:102863. 34700102 10.1016/j.nicl.2021.102863 PMC8551695

[37] Mao J, Zeng W, Zhang Q, Yang Z, Yan X, Zhang H, Wang M, Yang G, Zhou M, Shen J. Differentiation between high-grade gliomas and solitary brain metastases: a comparison of five diffusion-weighted MRI models. BMC Med Imaging. 2020;20(1):124. 33228564 10.1186/s12880-020-00524-w PMC7684933

[38] Horn A. A structural group-connectome in standard stereotactic (MNI) space. Data brief. 2015;5:292–6. 26543893 10.1016/j.dib.2015.08.035 PMC4589797

[39] Jenkinson M, Beckmann CF, Behrens TE, Woolrich MW, Smith SM. FSL. NeuroImage. 2012;62(2):782–90. 21979382 10.1016/j.neuroimage.2011.09.015

[40] Xie S, Chen L, Zuo N, Jiang T. DiffusionKit: A light one-stop solution for diffusion MRI data analysis. J Neurosci Methods. 2016;273:107–19. 27568099 10.1016/j.jneumeth.2016.08.011

[41] Tabesh A, Jensen JH, Ardekani BA, Helpern JA. Estimation of tensors and tensor-derived measures in diffusional kurtosis imaging. Magn Reson Med. 2011;65(3):823–36. 21337412 10.1002/mrm.22655 PMC3042509

[42] Yan X, Zhou M, Ying L, Liu W, Yang G, Wu D, Zhou Y, Peterson BS, Xu D. A fast schema for parameter estimation in diffusion kurtosis imaging. Comput Med imaging graphics: official J Comput Med Imaging Soc. 2014;38(6):469–80. 10.1016/j.compmedimag.2014.06.010 PMC4150694 25016957

[43] Daducci A, Canales-Rodríguez EJ, Zhang H, Dyrby TB, Alexander DC, Thiran JP. Accelerated Microstructure Imaging via Convex Optimization (AMICO) from diffusion MRI data. NeuroImage. 2015;105:32–44. 25462697 10.1016/j.neuroimage.2014.10.026

[44] McDougall F, Edgar C, Mertes M, Delmar P, Fontoura P, Abi-Saab D, Lansdall CJ, Boada M, Doody R. Psychometric Properties of the Clinical Dementia Rating - Sum of Boxes and Other Cognitive and Functional Outcomes in a Prodromal Alzheimer’s Disease Population. J Prev Alzheimer’s disease. 2021;8(2):151–60. 33569561 10.14283/jpad.2020.73

[45] Terhoeven V, Faschingbauer S, Huber J, Herzog W, Friederich HC, Simon JJ, Nikendei C. Verbal memory following weight gain in adult patients with anorexia nervosa: A longitudinal study. Eur Eat disorders review: J Eat Disorders Association. 2023;31(2):271–84. 10.1002/erv.2956 36397677

[46] Balthazar ML, Yasuda CL, Cendes F, Damasceno BP. Learning, retrieval, and recognition are compromised in aMCI and mild AD: are distinct episodic memory processes mediated by the same anatomical structures? J Int Neuropsychological Society: JINS. 2010;16(1):205–9. 10.1017/S1355617709990956 19835661

[47] Berg L. Clinical Dementia Rating (CDR). Psychopharmacol Bull. 1988;24(4):637–9. 3249765

[48] Desikan RS, Ségonne F, Fischl B, Quinn BT, Dickerson BC, Blacker D, Buckner RL, Dale AM, Maguire RP, Hyman BT, et al. An automated labeling system for subdividing the human cerebral cortex on MRI scans into gyral based regions of interest. NeuroImage. 2006;31(3):968–80. 16530430 10.1016/j.neuroimage.2006.01.021

