# Supplementary material for: Neurite orientation dispersion and density imaging identifies microstructural alterations in patients with cerebral microbleeds and correlations with cognitive impairment
Source: BMC Neurol. 2026 May 23;26:480. doi: 10.1186/s12883-026-04994-3 (PMC13412129; doi:10.1186/s12883-026-04994-3)
Supplement: Supplementary file 1 — Supplementary Material 1. [file 12883_2026_4994_MOESM1_ESM.docx]

**Supplementary Material**

Supplementary Table 1: Cerebral Clusters with Significantly Altered NODDI Metrics (ICVF and ISOVF) after Adjusting for Cognitive Diagnosis (CN/MCI) (voxel-wise between-group comparisons, FDR corrected, P < 0.05)

| NODDI metrics | Cluster size | Peak voxel | Regions |
| --- | --- | --- | --- |
|  |  | MNI Coordinates (mm) |  |
|  |  | x y z |  |
| ICVF (CSVD-c < HC) | 337 | 22 20 12 | Right caudate |
|  | 281 | -20 20 14 | Left caudate |
| ISOVF (CSVD-c > HC) | 264 | 10 -62 48 | Right precuneus |
|  | 218 | -26 -74 24 | Left superior parietal |
| ICVF (CSVD-c < CSND-n) | 288 | -14 10 12 | Left caudate |
|  | 213 | 12 8 8 | Right caudate |

Notes: Anatomical localizations of peak MNI coordinates were established according to the cortical Desikan atlas and subcortical FreeSurfer ASEG atlases.

Abbreviations: ICVF, Intracellular Volume Fraction; ISOVF, Isotropic Volume Fraction; CSVD, Cerebral small vessel disease; CSVD-n, CSVD without CMBs; CSVD-c, CSVD with CMBs; CMBs, Cerebral microbleeds; HC, Healthy controls. FDR, False discovery rate.
